# Clinical presentation and outcomes in non-maltophilia *Stenotrophomonas* spp. bloodstream infections

**DOI:** 10.1038/s41598-026-59102-9

**Published:** 2026-07-06

**Authors:** Matthew Chung Yi Koh, Jinghao Nicholas Ngiam, Jeanette Teo, Ka Lip Chew

**Affiliations:** 1 Division of Infectious Diseases, Department of Medicine, National University Health System, Singapore, Singapore; 2 Department of Laboratory Medicine, National University Hospital, Singapore, Singapore

**Keywords:** *Stenotrophomonas maltophilia*, Whole genome sequencing, Genomic diversity, Clinical outcomes, Bacteraemia, Diseases, Microbiology

## Abstract

*Stenotrophomonas maltophilia* complex is an important nosocomial cause of bacteraemia. The complex comprises multiple species, but their relative clinical and genomic significance remains unclear. We examined clinical and genomic differences between *S. maltophilia* and non-*S. maltophilia* species to better understand their virulence and implications for patient care. Hospitalized adult patients (≥ 21 years) between January 2022 and June 2024 with *Stenotrophomonas* species bacteraemia were examined. Clinical data were extracted from electronic medical records. Whole-genome sequencing was performed on all isolates, with species assignment confirmed by average nucleotide identity. *S. maltophilia* and non-*S. maltophilia* species were compared. Fifty-three *S. maltophilia* complex isolates were identified, of which 45 had clinical data. *S. maltophilia* predominated (n = 32), followed by *S. pavanii* (n = 7), *S. sepilia* (n = 4), *S. muris* (n = 1), and *S. geniculata* (n = 1). *S. maltophilia* isolates formed multiple clades with diverse sequence types, consistent with both clonal expansion and ongoing diversification. Virulence genes, including *stmPr*, *smoR*, and iron acquisition-associated genes were conserved across *S. maltophilia* lineages, but *stmPr1* was less frequently observed in non-*S. maltophilia*. Most infections were line-related. Pneumonia-associated bacteraemia appeared more frequently among with *S. maltophilia* infections. Polymicrobial bacteraemia appeared more commonly in non-*S. maltophilia* infections. Mortality was similar between groups, but recurrence of infection within one year occurred only in *S. maltophilia*. Compared with non-*S. maltophilia, S. maltophilia* appeared to be associated with pneumonia and recurrent bacteraemia episodes, although mortality was similar. These exploratory findings suggest potential differences within the complex, but require validation in larger studies.

## Introduction

*Stenotrophomonas maltophilia* complex has emerged as an important nosocomial pathogen in healthcare settings, particularly among immunocompromised and critically ill patients.^1,2^ Historically regarded as a low-virulence environmental organism, *S. maltophilia* is now recognised as a significant cause of nosocomial bloodstream infections and pneumonia, often in association with indwelling devices and prior broad-spectrum antibiotic exposure.^1^ Its intrinsic multidrug resistance, biofilm-forming capacity, and array of virulence determinants, such as quorum-sensing regulators, secreted proteases, and iron-acquisition system may pose an increasing challenge for therapy and infection control.^3–5^ However, accumulating genomic evidence indicates that *S. maltophilia* represents a diverse complex of related species with variable pathogenic potential, yet their clinical relevance remains poorly defined.

Clarifying whether the species-level genomic diversity within the *S. maltophilia* complex translates into distinct clinical phenotypes may guide clinical management, as well as inform appropriate infection control measures and epidemiological surveillance. Whole genome sequencing allows for accurate delineation of *Stenotrophomonas* species, in addition to antimicrobial resistance and virulence genes.^6^ In this study, we examined bloodstream infections due to members of the *S. maltophilia* complex at a tertiary hospital in Singapore, integrating clinical, phenotypic, and genomic analyses to explore species distribution, virulence gene profiles, in association with the clinical presentation and outcomes. By comparing *S. maltophilia* with non-*S. maltophilia* species, we aimed to identify potential differences in pathogenicity, in order to refine our understanding of this organism and its implications for patient care.

## Methods

We conducted a single-centre retrospective cohort study of consecutive adult patients (≥ 21 years) admitted to the National University Hospital, Singapore, between 1 January 2022 and 30 June 2024 who had at least one positive blood culture for *Stenotrophomonas* species. Patients were identified through the hospital microbiology database, and relevant clinical data were extracted from the electronic medical records. Demographic characteristics, comorbidities, immunocompromising conditions, source of infection, antimicrobial exposure, and clinical outcomes were recorded. Nosocomial onset was defined as bacteraemia occurring > 48 h after admission, and all-cause in-hospital mortality was used as the primary outcome measure.

Microbiological identification was performed using matrix-assisted laser desorption/ionization time-of-flight mass spectrometry (MALDI-TOF MS; Bruker Biotyper). Routine antimicrobial susceptibility testing employed Etest strips for trimethoprim-sulfamethoxazole, levofloxacin, and minocycline in accordance with institutional protocol. All isolates were initially identified as *S. maltophilia* by MALDI-TOF MS (Bruker Biotyper).

Whole genome sequencing (WGS) was performed using the Illumina NovaSeq 6000 platform (Illumina Inc., San Diego, CA, USA), generating 150-bp paired-end reads. FastQC to evaluate read quality (https://www.bioinformatics.babraham.ac.uk/projects/fastqc/). Sequencing reads were assembled using Shovill (https://github.com/tseemann/shovill). Assembled genomes were assessed for completeness using BUSCO with the bacteria_odb10 database.^7^ Core genome SNP phylogenetic analysis was conducted using CSI Phylogeny (https://cge.food.dtu.dk/services/CSIPhylogeny/). Tree visualization and annotation were performed using Interactive Tree Of Life (iTOL).^8^ Genome annotation was performed using Prokka.^9^ ABRicate (https://github.com/tseemann/abricate) and Resfinder (http://genepi.food.dtu.dk/resfinder) were used to screen antimicrobial resistance (AMR) determinants. AMR genes were identified using a threshold of > 98% identity to their respective database matches. Sequence types (STs) were determined with PubMLST Stenotrophomonas maltophilia (https://pubmlst.org/organisms/stenotrophomonas-maltophilia). Average nucleotide identity (ANI) analysis was performed using FastANI^10^ against publicly available Stenotrophomonas reference genomes obtained from the NCBI database (https://www.ncbi.nlm.nih.gov/datasets/genome/?taxon=40323). Species assignment was based on a 96% ANI threshold for bacterial species delineation.^11^.

For statistical analysis, patients were stratified into two groups: those infected with *S. maltophilia* and those with non-*S. maltophilia* species (including *S. pavanii*, *S. sepilia*, *S. geniculata*, and *S. muris*). Continuous variables were compared using Student’s t-test and expressed as mean ± standard deviation, while categorical variables were compared using the chi-square or Fisher’s exact test as appropriate. A two-tailed *p* value < 0.05 was considered statistically significant. Analyses were performed using SPSS version 20.0 (SPSS Inc., Chicago, IL). This study was conducted in accordance with the Declaration of Helsinki, and was approved by the National Healthcare Group Domain-Specific Review Board (DSRB 2024–3132) with a waiver of informed consent as only de-identified data were analysed.

## Results

**Fig. 1:**
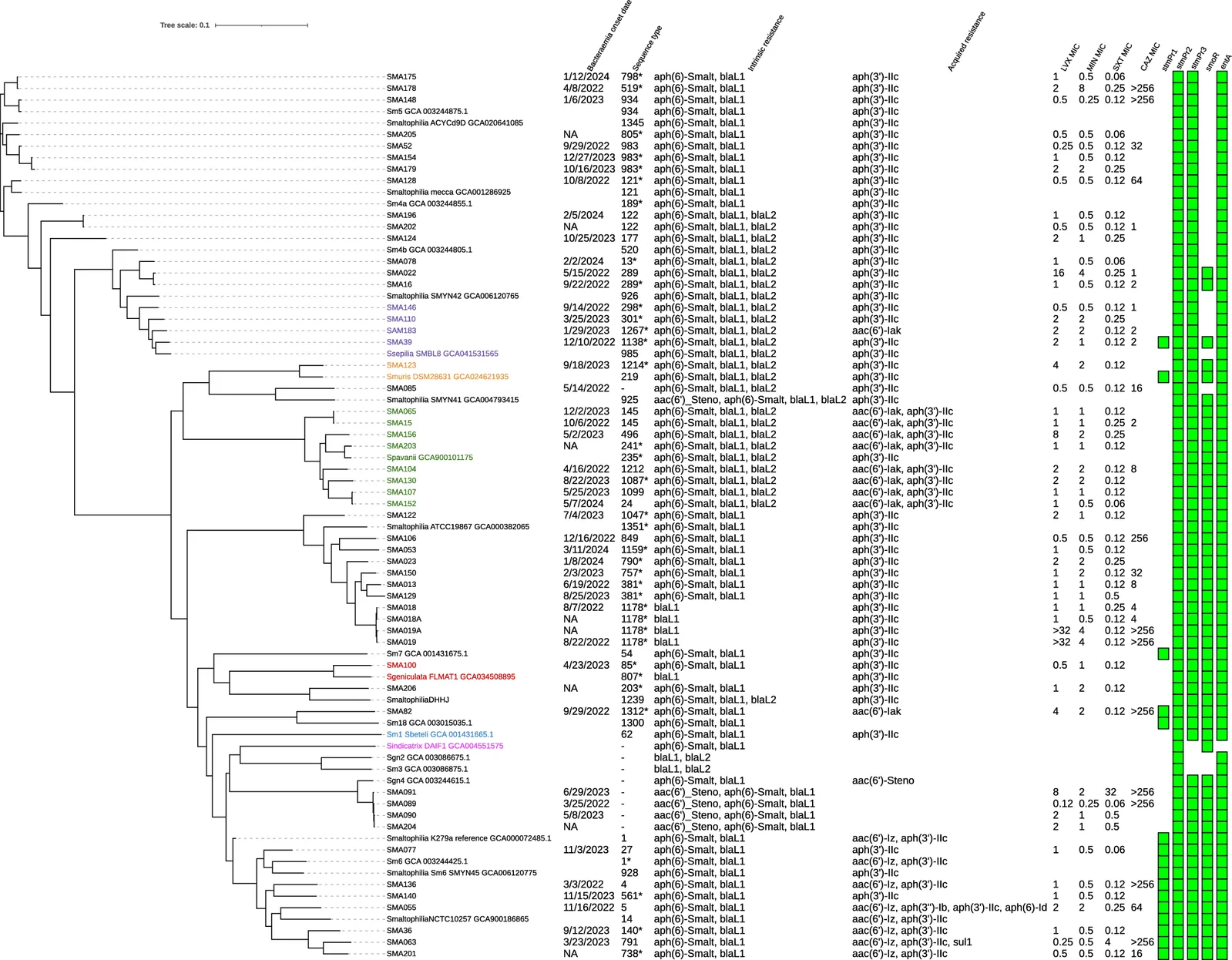
Core genome SNP phylogenetic tree of *Stenotrophomonas maltophilia* complex isolates annotated with sequence type, intrinsic and acquired antimicrobial resistance genes, antimicrobial susceptibility profiles including levofloxacin (LVX), minocycline (MIN), trimethoprim-sulfamethoxazole (SXT), and ceftazidime (CAZ) MICs, and selected virulence-associated genes including stmPr1 (zinc metalloprotease), stmPr2 (metalloprotease), stmPr3 (serine protease), smoR (quorum-sensing regulator), and entA (enterobactin biosynthesis enzyme). Publicly available reference genomes and their corresponding NCBI accession numbers were included in the analysis. Closest ST matches are indicated with an asterisk (*). Isolate names in different colour font indicate different species: Black: *S. maltophilia*; Purple: *S. sepilia*; Orange: *S. muris*; Green: *S. pavanii*; Red: *S. geniculata*.

Illumina NovaSeq sequencing generated reads with an average sequencing depth of approximately 300 × across all isolates. Genome assemblies demonstrated high completeness, with BUSCO completeness scores exceeding 99%. Core genome SNP phylogenetic analysis (Fig. 1), together with ANI analysis supported species assignment. A total of 53 *Stenotrophomonas maltophilia* complex isolates were identified, of which 45 had complete clinical data available for analysis. *S. maltophilia* was the predominant species (n = 32, 71%), followed by *S. pavanii* (n = 7), *S. sepilia* (n = 4), *S. muris* (n = 1), and *S. geniculata* (n = 1). The mean age was 64 ± 14 years, and 62% were male. Most patients were immunocompromised (60%) and had central venous catheters in situ (84%). Infections were primarily nosocomial in onset (76%) and commonly associated with indwelling devices. The majority of episodes were line-related infections (64%), followed by pneumonia (16%).

The overall distribution of infection sources differed between *S. maltophilia* and *non-S. maltophilia* species (*p* < 0.001). In particular, pneumonia as the source appeared more common with S*. maltophilia* infections (6/32, 18.8%) than non-*S. maltophilia* infections 1/13 (7.7%), although this individual category was interpreted descriptively given the small number of events across each source category. Concomitant bacteraemia with other pathogens were seen in both groups, but appeared more common in non-*S. maltophilia* infections (62% vs 32%, *p* = 0.098), with a trend towards statistical significance. Baseline comorbidities, haemodynamic parameters, and inflammatory markers were similar between groups, though hyperlipidaemia was more common among patients with *S. maltophilia* bacteraemia (50% vs 15%, *p* = 0.045). Intervention for source control of infection (e.g. removal of infected lines) was achieved in a majority of cases overall, and in-hospital mortality did not differ significantly between *S. maltophilia* and non-*S. maltophilia* species (34% vs 46%, *p* = 0.460). Recurrence of bacteraemia within one year was observed only among *S. maltophilia* cases, although the number of recurrent episodes was small and did not reach statistical significance. Additionally, genomic confirmation of relapse versus reinfection was not available for all paired isolates, limiting further characterization of these episodes (Table 1). Genomic analysis demonstrated substantial diversity among *S. maltophilia* complex isolates, with multiple sequence types distributed across distinct phylogenetic clades without a single dominant lineage associated with specific clinical syndromes in this cohort.

**Table 1:** Clinical characteristics of patients with *Stenotrophomonas maltophilia* complex bloodstream infections by species.

Parameter	*S. maltophilia* (n = 32)	Non-*S. maltophilia* species* (n = 13)	*P*-value
Age (years)	65 (± 13)	61 (± 16)	0.356
Sex (male)	20 (62.5%)	8 (61.5%)	0.952
Hypertension	17 (53.1%)	5 (38.5%)	0.372
Hyperlipidaemia	16 (50.0%)	2 (15.4%)	0.045
Diabetes mellitus	13 (40.6%)	4 (30.8%)	0.537
End-stage kidney disease	6 (18.8%)	2 (15.4%)	0.789
Immunocompromising condition	19 (59.4%)	8 (61.5%)	0.893
Presence of central line	26 (81.3%)	12 (92.3%)	0.354
Source of infection			< 0.001
Central line-related	20 (62.5%)	9 (69.2%)	
Pneumonia	6 (18.8%)	1 (7.7%)	
Skin and soft tissue	1 (3.1%)	0 (0.0%)	
Intra-abdominal	4 (12.5%)	2 (15.4%)	
Urinary tract infection	1 (3.1%)	0 (0.0%)	
Others	0 (0.0%)	1 (7.7%)	
Nosocomial onset	24 (75.0%)	10 (76.9%)	0.892
Intensive care onset	9 (28.1%)	6 (46.2%)	0.245
Prior carbapenem exposure	18 (56.3%)	8 (61.5%)	0.745
Concomitant bacteraemia with other organisms	10 (32.3%)	8 (61.5%)	0.098
Required inotropic support	4 (12.5%)	1 (7.7%)	0.642
Systolic blood pressure (mmHg)	118 (± 29)	110 (± 15)	0.964
Total white cell count (× 10^9^/L)	10.0 (± 7.6)	8.9 (± 7.8)	0.659
C-reactive protein (mg/L)	154 (± 189)	168 (± 231)	0.412
Serum creatinine (µmol/L)	106 (± 82)	131 (± 111)	0.841
Source control of infection obtained (e.g. removal of infected central line, drainage of abscess)	25 (78.1%)	9 (69.2%)	0.529
Mortality	11 (34.4%)	6 (46.2%)	0.460
Recurrence of bacteraemia in 1 year	4 (12.5%)	0 (0.0%)	0.182

*Non-*S. maltophilia* isolates were identified as: *S. pavani* (n = 7), *S. sepilia* (n = 4), *S. geniculata* (n = 1), *S. muris* (n = 1).

Among the 53 sequenced isolates, intrinsic resistance determinants were common, with *blaL1* present in all isolates (53/53, 100%), *aph(6)-Smalt* in 49/53 (92.5%), and *blaL2 i*n 20/53 (37.7%). The aminoglycoside resistance gene *aph(3’)-IIc* was detected in 47/53 (88.7%) isolates, while other acquired resistance genes were less frequent, including *aac(6’)-Iak* in 10/53 (18.9%), *aac(6’)-Iz* in 5/53 (9.4%), and *aac(6’)_Steno* in 4/53 (7.5%). Phenotypic minimum inhibitory concentrations (MICs) ranged from 0.12 to > 32 mg/L for levofloxacin, and 1 to > 256 mg/L for ceftazidime. Minocycline (0.25–8 mg/L) and trimethoprim-sulfamethoxazole (0.06–32 mg/L) MICs generally remained low across isolates. Among selected virulence-associated genes, *stmPr2*, *stmPr3*, and *entA* were present in all sequenced isolates, while *smoR* was detected in 37/53 (69.8%) and *stmPr1* in 9/53 (17.0%). *stmPr1* was less frequently observed among non-*S. maltophilia* isolates than *S. maltophilia* isolates, although the small number of non-*S. maltophilia* isolates limits interpretation (Fig. 1).

## Discussion

In this single-centre study of *Stenotrophomonas maltophilia* complex bloodstream infections, *S. maltophilia* emerged as the predominant species and appeared to differ clinically and genomically from other members of the complex. While both *S. maltophilia* and non-*S. maltophilia* species primarily caused line-related bacteraemia in immunocompromised hosts, pneumonia as a source of infection was observed more frequently among *S. maltophilia* cases. Hyperlipidaemia was more frequently observed among patients with *S. maltophilia* bacteraemia in this cohort, however, given the limited sample size and multiple comparisons performed, the clinical significance of this finding remains uncertain.

Although recurrent bacteraemia episodes were observed only among *S. maltophilia* cases in this cohort, the small number of events and lack of complete genomic comparison between paired isolates limit definitive distinction between relapse and reinfection. All patients received culture-directed antimicrobial therapy, although antibiotic selection and duration were determined by the treating physicians in this retrospective uncontrolled study.

Genomic analysis provided complementary insights into these clinical patterns. Conserved virulence determinants, such as the metalloprotease *stmPr*, the quorum-sensing regulator *smoR*, and iron-acquisition systems probably contribute to its pathogenicity.^13^ Non-*S. maltophilia* isolates appeared to carry the virulence-associated gene *stmPr1* less frequently. However, all members of the *S. maltophilia* complex should still be regarded as opportunistic pathogens, and the clinical significance of differences in virulence-associated genes remains uncertain.^12^ The diversity of *S. maltophilia* sequence types suggests both clonal persistence and ongoing diversification within the hospital environment. No specific sequence type or phylogenetic lineage was clearly associated with particular clinical syndromes or outcomes in this cohort. The widespread presence of intrinsic β-lactamase genes such as *blaL1* and *blaL2* across isolates is consistent with the known intrinsic resistance profile of the *S. maltophilia* complex. However, phenotypic susceptibility patterns remained variable despite conserved resistance determinants, which was likely representative of the complexity of genotype–phenotype relationships and the limitations of predicting antimicrobial susceptibility solely from genomic data.

Overall, although *S. maltophilia* isolates in this cohort had more frequently presented with bacteraemia from pneumonia, with episodes of recurrent bacteraemia, and a higher prevalence of the virulence-associated gene *stmPr1*, these findings did not translate into higher in-hospital mortality. Mortality in bloodstream infections is likely influenced by multiple host and clinical factors beyond organism-specific genomic characteristics alone. Recent clinical cohort studies of *S. maltophilia* bacteraemia have similarly reported substantial mortality, with outcomes influenced by host factors, illness severity, adequacy of source control, and choice of antimicrobial therapy.^1,2,14^ These findings are consistent with our observation that mortality was unlikely to be driven by species identity alone. In our cohort, the difference in mortality between *S. maltophilia and* non-*S. maltophilia* had not been statistically significant and should therefore be interpreted cautiously. Accordingly, the observed genomic differences should not be interpreted as direct evidence of increased virulence, as it did not translate to statistically higher mortality, particularly as functional virulence phenotyping was also not performed in this study.

The study was also limited by its retrospective design and relatively small sample size, particularly within the non-*S. maltophilia* subgroup, which limited statistical power and the ability to draw definitive conclusions regarding clinical differences between species. However, the study highlights important potential distinctions within the *S. maltophilia* complex that warrant further investigation. Larger multicentre studies integrating genomic and transcriptomic data will be essential to delineate pathogenic mechanisms and guide targeted management strategies for this emerging nosocomial pathogen.

## Conclusions

Our findings highlight potential clinical and genomic differences between different species within the *Stenotrophomonas maltophilia* complex. *S. maltophilia* may be more associated with pneumonia and recurrent bacteraemia episodes, but mortality remained similar. Larger studies are required to determine whether these observations translate into meaningful differences in clinical outcomes. Future studies in *S. maltophilia* complex infections should include species-level identification and sub-analysis to further characterize these potential differences.

## Data Availability

The sequencing data generated in this study have been deposited in the NCBI Sequence Read Archive under BioProject accession number PRJNA1453040 (Submission ID: SUB16119178).

## References

[1] Koh, M. C. Y. et al. Clinical presentation and outcomes of bloodstream infection with intrinsically carbapenem-resistant non-fermenting gram-negative organisms: *Stenotrophomonas maltophilia*, *Elizabethkingia* spp. and *Chryseobacterium* spp. in Singapore, from 2012 to 2024. *BMC Infect Dis* **25**(1), 273. 10.1186/s12879-025-10636-9 (2025). 40000958 PMC11863829

[2] Koh, M. C. Y. et al. Risk factors for mortality and implications on therapy for Stenotrophomonas maltophilia bacteraemia. *J. Infect. Public Health* **18**(8), 102829. 10.1016/j.jiph.2025.102829 (2025). 40409223

[3] Crossman, L. C. et al. The complete genome, comparative and functional analysis of *Stenotrophomonas**maltophilia* reveals an organism heavily shielded by drug resistance determinants. *Genome Biol.* **9**(4), R74. 10.1186/gb-2008-9-4-r74 (2008). 18419807 PMC2643945

[4] Azimi, A. et al. Emergence of fluoroquinolone resistance and possible mechanisms in clinical isolates of *Stenotrophomonas**maltophilia* from Iran. *Sci. Rep.* **11**(1), 9582. 10.1038/s41598-021-88977-z (2021). 33953262 PMC8100118

[5] Banar, M. et al. Global prevalence and antibiotic resistance in clinical isolates of *Stenotrophomonas**maltophilia*: A systematic review and meta-analysis. *Front. Med.* **10**, 1163439. 10.3389/fmed.2023.1163439 (2023). PMC10196134 37215718

[6] Shahid, S. et al. Antibiotic resistance genes prediction via whole genome sequence analysis of *Stenotrophomonas**maltophilia*. *J. Infect. Public Health* **17**(2), 236–244. 10.1016/j.jiph.2023.12.010 (2024). 38128408

[7] Simão, F. A., Waterhouse, R. M., Ioannidis, P., Kriventseva, E. V. & Zdobnov, E. M. BUSCO: Assessing genome assembly and annotation completeness with single-copy orthologs. *Bioinformatics* **31**(19), 3210–3212. 10.1093/bioinformatics/btv351 (2015). 26059717

[8] Letunic, I. & Bork, P. Interactive tree of life (iTOL) v3: An online tool for the display and annotation of phylogenetic and other trees. *Nucleic Acids Res.* **44**(W1), W242-245. 10.1093/nar/gkw290 (2016). 27095192 PMC4987883

[9] Seemann, T. Prokka: rapid prokaryotic genome annotation. *Bioinformatics* **30**(14), 2068–2069. 10.1093/bioinformatics/btu153 (2014). 24642063

[10] Jain, C., Rodriguez-R, L. M., Phillippy, A. M., Konstantinidis, K. T. & Aluru, S. High throughput ANI analysis of 90K prokaryotic genomes reveals clear species boundaries. *Nat. Commun.* **9**(1), 5114. 10.1038/s41467-018-07641-9 (2018). 30504855 PMC6269478

[11] Richter, M. & Rosselló-Móra, R. Shifting the genomic gold standard for the prokaryotic species definition. *Proc. Natl. Acad. Sci. U. S. A.* **106**(45), 19126–19131. 10.1073/pnas.0906412106 (2009). 19855009 PMC2776425

[12] Wang, L. et al. Molecular epidemiology, genetic diversity, antibiotic resistance and pathogenicity of *Stenotrophomonas**maltophilia* complex from bacteremia patients in a tertiary hospital in China for nine years. *Front. Microbiol.* **15**, 1424241. 10.3389/fmicb.2024.1424241 (2024). 38946894 PMC11211261

[13] Bhaumik, R., Aungkur, N. Z. & Anderson, G. G. A guide to *Stenotrophomonas**maltophilia* virulence capabilities, as we currently understand them. *Front. Cell. Infect. Microbiol.* **13**, 1322853. 10.3389/fcimb.2023.1322853 (2023). 38274738 PMC10808757

[14] Sezen, A. I. et al. Seven-year evaluation of *Stenotrophomonas**maltophilia* bacteremia in a university-affiliated hospital. *J. Infect. Dev. Ctries.* **19**(4), 498–503. 10.3855/jidc.20243 (2025). 40305526

